# The association between heat exposure and hospitalization for undernutrition in Brazil during 2000−2015: A nationwide case-crossover study

**DOI:** 10.1371/journal.pmed.1002950

**Published:** 2019-10-29

**Authors:** Rongbin Xu, Qi Zhao, Micheline S. Z. S. Coelho, Paulo H. N. Saldiva, Michael J. Abramson, Shanshan Li, Yuming Guo

**Affiliations:** 1 Department of Epidemiology, School of Public Health and Management, Binzhou Medical University, Yantai, Shandong, China; 2 Department of Epidemiology and Preventive Medicine, School of Public Health and Preventive Medicine, Monash University, Melbourne, Victoria, Australia; 3 Institute of Advanced Studies, University of São Paulo, São Paulo, Brazil; St. Michael's Hospital, CANADA

## Abstract

**Background:**

Global warming is predicted to indirectly result in more undernutrition by threatening crop production. Whether temperature rise could affect undernutrition directly is unknown. We aim to quantify the relationship between short-term heat exposure and risk of hospitalization due to undernutrition in Brazil.

**Methods and findings:**

We collected hospitalization and weather data for the hot season (the 4 adjacent hottest months for each city) from 1,814 Brazilian cities during 1 January 2000−31 December 2015. We used a time-stratified case-crossover design to quantify the association between heat exposure and hospitalization due to undernutrition. Region-specific odds ratios (ORs) were used to calculate the attributable fractions (AFs). A total of 238,320 hospitalizations for undernutrition were recorded during the 2000−2015 hot seasons. Every 1°C increase in daily mean temperature was associated with a 2.5% (OR 1.025, 95% CI 1.020−1.030, *p <* 0.001) increase in hospitalizations for undernutrition across lag 0–7 days. The association was greatest for individuals aged ≥80 years (OR 1.046, 95% CI 1.034−1.059, *p <* 0.001), 0–4 years (OR 1.039, 95% CI 1.024–1.055, *p <* 0.001), and 5–19 years (OR 1.042, 95% CI 1.015–1.069, *p =* 0.002). Assuming a causal relationship, we estimate that 15.6% of undernutrition hospitalizations could be attributed to heat exposure during the study period. The AF grew from 14.1% to 17.5% with a 1.1°C increase in mean temperature from 2000 to 2015. The main limitations of this study are misclassification of different types of undernutrition, lack of individual temperature exposure data, and being unable to adjust for relative humidity.

**Conclusions:**

Our study suggests that global warming might directly increase undernutrition morbidity, by a route other than by threatening food security. This short-term effect is increasingly important with global warming. Global strategies addressing the syndemic of climate change and undernutrition should focus not only on food systems, but also on the prevention of heat exposure.

## Abstract 2

Rongbin Xu and colleagues reveal the association between heat exposure and hospital admissions for undernutrition in Brazil.

## Author summary

**Why was this study done?:**

- It has been well documented that global warming will indirectly result in more undernourished people by threatening crop production in the long term.
- However, no study has yet evaluated the short-term and direct effect of heat exposure on morbidity related to undernutrition.

**What did the researchers do and find?:**

- With daily hospitalization data covering nearly 80% of the Brazilian population during 2000−2015, we used a time-stratified case-crossover design to quantify the association between heat exposure and hospitalization for undernutrition. We also estimated the heat-related hospitalization burden.
- There was a 2.5% increase in undernutrition hospitalizations following every 1°C increase in daily mean temperature during the hot season. The young (0–19 years) and elderly people (≥80 years) with undernutrition were more vulnerable to heat exposure than other age groups.
- Heat exposure was responsible for 15.6% of the undernutrition hospitalizations during the study period, corresponding to 37,129 cases. The attributable fraction increased from 14.1% in 2000 to 17.5% in 2015, paralleling a 1.1°C increase in average temperature.

**What do these findings mean?:**

- Rising temperature might impact undernutrition morbidity directly and more rapidly than through threatening food security alone.
- The possible pathways of this direct impact of heat might include reducing undernourished people’s food intake, impairing their digestion and absorption function, and causing fluid and electrolyte disturbances.
- This direct, short-term effect will be increasingly important with global warming, as illustrated by what happened in Brazil over 2000−2015.
- Global strategies addressing the syndemic of climate change and undernutrition should focus not only on food systems, but also on the prevention of heat exposure, especially among the young and elderly.

## Introduction

Undernutrition means inadequate intake of energy and nutrients to meet an individual’s needs to maintain good health [1]. Despite huge nutritional improvement in recent decades, undernutrition remains a big global public health concern, especially in low- and middle-income countries (LMICs). In 2016, about 420 million adults aged 20 years or above and 192 million children and adolescents aged 5–19 years worldwide were underweight, and nearly 90% of them resided in LMICs [2]. There were 150.8 million children under 5 years old who were stunted and 51 million who were wasted in 2017 [3]. Around 45% of deaths among children under 5 years old are associated with undernutrition [4].

Recently, there has been an increasing interest in the “syndemic” (synergy of epidemics) of climate change and undernutrition [5–7]. It is anticipated that global climate change, with increasing temperature and more extreme rainfall, will reduce future crop yields and threaten food security, thus potentially resulting in more undernutrition [8–10]. Some researchers have estimated that climate change will lead to a 1%–29% increase in moderate stunting and a 23%–62% increase in severe stunting by 2050 [8]. However, current studies all focus on the long-term, indirect impact of climate change on undernutrition. No previous study, to our knowledge, has evaluated the short-term, direct effect of climate factors—specifically temperature rise—on undernutrition morbidity [5–7,9,10].

In this study, we characterize the association between short-term heat exposure and risk of hospitalization due to undernutrition, using a national hospitalization dataset spanning 2000–2015 in Brazil. Specifically, this study examines the impact of heat exposure on the hospitalization of individuals with existing malnutrition, rather than the heat causing malnutrition de novo over very short time frames. Further, we explore whether the association is consistent across types of undernutrition and across subgroups of the population based on age, sex, and region. Finally, assuming a causal relationship, we estimate the fraction of all hospitalizations for undernutrition that were attributable to heat exposure.

## Methods

This time-stratified case-crossover study is reported following the REporting of studies Conducted using Observational Routinely-collected health Data (RECORD) statement (S1 RECORD Checklist) [11]. We performed the data analyses for this study according to a prospective analysis plan (S1 Text). Modifications to the analysis plan are also described in S1 Text.

### Data collection

**Fig 1:**
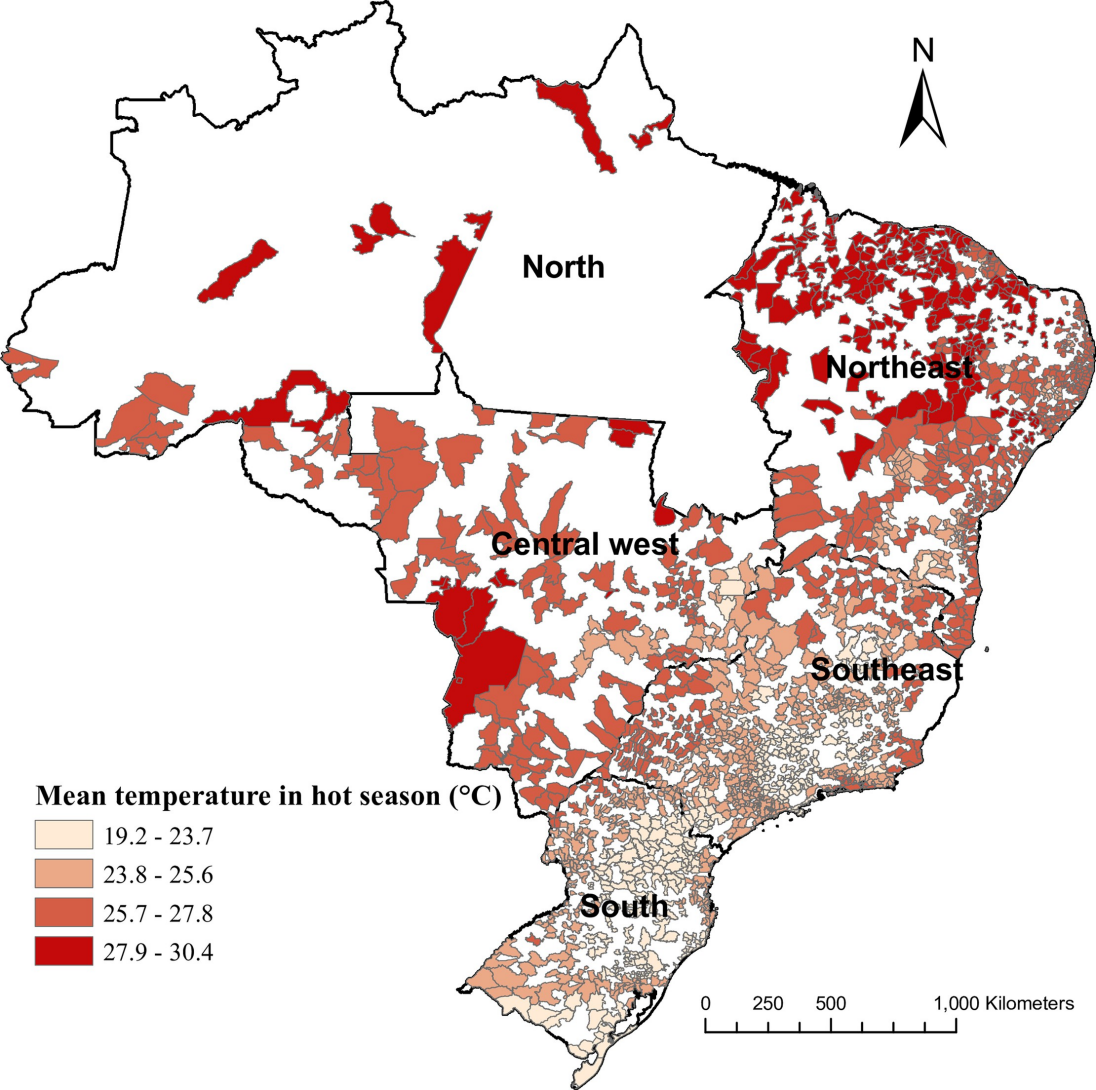
Location of the included 1,814 cities in Brazil and their daily mean temperature in the hot season during 2000−2015. The base map of this figure was downloaded from the Brazilian Institute of Geography and Statistics (https://www.ibge.gov.br/); the base map was free and open-access.

We collected hospital admission data from Brazilian Unified Health System (BUHS) from 1 January 2000 to 31 December 2015. The authors had access to the full hospitalization data recorded by BUHS during the study period. The full dataset covered 5,570 cities. However, to minimize the effects of missing values, we only included data from the 1,814 cities with complete hospitalization records over the 16 years. These cities comprised 78.4% of the national population, and were distributed in 5 regions of Brazil (North, Northeast, Central West, Southeast, and South) (Fig 1) [12,13]. Information such as date of admission, primary diagnosis, sex, and age for each hospital admission was recorded by BUHS. The primary diagnosis was coded according to the International Classification of Diseases–10th revision (ICD-10). We extracted hospitalization data with ICD-10 codes for undernutrition (E40–E46; see https://icd.who.int/browse10/2016/en). As a result, we only analyzed hospitalizations whose primary cause was undernutrition. To assist subgroup analyses, we classified undernutrition into 5 categories: severe protein–energy malnutrition (PEM) (ICD-10 code E40–E43), moderate PEM (E44.0), mild PEM (E44.1), retarded development following PEM (E45), and unspecified PEM (E46). Ethics approval was not required for our analysis of aggregate anonymized data from the BUHS.

We obtained the daily minimum and maximum temperatures from a national meteorological dataset (0.25° × 0.25° resolution) developed by Xavier et al. [14]. The dataset was interpolated by inverse distance weighting using data from 735 weather stations in Brazil. We used the weather of the city center to represent each city. In this study, daily mean temperature (as approximated by the average of the daily minimum and maximum temperatures) during the hot season was used to represent heat exposure [13]. Daily weather data were linked to hospitalization cases according to city and date.

We also collected daily relative humidity recorded from city-specific weather stations by the Brazilian National Institute of Meteorology. Unfortunately, humidity data were available only for 193 cities and only during 2000−2012. Because most Brazilian cities do not have air quality monitoring stations, we were unable to get sufficient data on air pollution for modeling.

### Statistical analyses

#### Assessing the temperature–hospitalization association

Because this study focused on the effect of heat exposure, or high temperature, the analyses were restricted to the hot season (defined as the 4 adjacent hottest months for each city) [13]. We used a time-stratified case-crossover design with conditional logistic regression models to evaluate the association between hospitalization for undernutrition and heat exposure [15,16]. For each hospital admission, the daily mean temperatures during the risk period (i.e., for the admission date and 1 to 7 days before admission according to our preliminary analysis) were compared with those during control periods in the same city. For each case, the same days of the week in the same calendar month were selected as controls. In this design, each case had 3 or 4 control periods. This method for matching case and control is effective to control for time-dependent confounders (long-term trend, seasonality, and the effect of day of the week) and time-constant confounders (e.g., age, sex, income, and lifestyle) [17–19]. The time-stratified referent selection strategy has been proved to be the most efficient and unbiased strategy in a case-crossover design [17,20].

We used conditional logistic regression to fit the relationship between ambient temperature and risk of hospitalization, with the following equation [21,22]:

$$logit\;(P(case=1\;in\;stratum\;i|Temp,Holiday))=a_{stratum\;i}+cb(Temp)+\beta \times Holiday$$ (1)

In this equation, a stratum consists of 1 case (case = 1) and its 3 or 4 controls (case = 0), the total number of strata is equal to the number of hospitalizations for undernutrition; *P*(case = 1 in stratum *i*|Temp, Holiday) is the conditional probability of being a case in the *i*th stratum given the 2 independent variables Temp and Holiday; *a*_stratum *i*_ represents the constant or intercept of stratum *i* (each stratum has an intercept); Holiday is a binary variable indicating whether the date was a public holiday, and its coefficient β accounts for any changes in hospital use during public holidays; and cb(Temp) is a matrix produced by a cross-basis function for daily mean temperature modeled by a distributed lag linear or nonlinear model. A cross-basis function was used to model both the exposure–response relationship and the lag–response relationship at the same time. In each dimension, a specific smoothing function (e.g., linear function, natural cubic spline) can be used to define the shape of relationship [23,24]. To make the equation clear, we show it applied to example data in S1 Table.

We initially compared the model performance using a nonlinear model (natural cubic spline with 3 degrees of freedom [df]) and a linear model (linear function) in the exposure–response dimension. We found that the Bayesian information criterion (BIC) value of the linear model was lower than the BIC value of the nonlinear model (679604.2 versus 679640.4), and the relationship between temperature and hospitalization tended to be linear when using the nonlinear model (S1 Fig). This meant the linear model performed better than the nonlinear model for the exposure–response dimension [25]. Therefore, for the cb(Temp) of the final model, we used a linear function in the exposure–response dimension. We used a natural cubic spline with 3 df in the lag–response dimension (lag 0–7 days) in accordance with our previous study [13].

The heat exposure–hospitalization associations are presented as the odds ratio (OR) with 95% confidence interval (CI) of hospitalization for undernutrition associated with every 1°C increase in daily mean temperature. We performed stratified analyses by sex, age group (0–4, 5–19, 20–39, 40–59, 60–79, and ≥80 years), region, and type of undernutrition. We used random effect meta-regression fitted by the maximum likelihood method to check the statistical differences in the ORs between subgroups. Apart from 2 hospitalization cases that had missing values for sex, there were no other missing values in the hospitalization or weather dataset. Because the case-crossover design does not need to adjust for sex, the 2 cases with missing sex were kept in the model analyses, except for the subgroup analyses by sex.

#### Sensitivity analyses

Sensitivity analyses were conducted to check the robustness of our results. First, we changed the maximum number of lag days (from 7 days to 4, 5, 6, 8, 9, or 10 days) or the df of lag days (from 3 to 4) in the cross-basis function (see S2 Table). Second, we tried several models adjusting for the average relative humidity with lag 0–7 days using the dataset of 193 cities with data on relative humidity (see S3 Table). Third, we redefined the hot season to include the city-specific 5 or 6 adjacent hottest months (see S4 Table). Finally, we repeated our main model analyses in the cold months (city-specific 4 coldest months) and moderate months (city-specific months other than the cold months and hot season) (see S5 Table). The effect estimates from sensitivity analyses were compared to the effect estimates from our primary models. For the first 3 sensitivity analyses, we used fixed effect meta-regressions with no statistical adjustment to check whether the differences in effect estimates from different models were statistically significant, because those models were based on the same or overlapping samples [26]. For the fourth sensitivity analysis, we used random effect meta-regression fitted by the maximum likelihood method to compare the effect estimates, because results in different seasons were based on different samples.

#### Calculating the attributable burden of hospitalization for undernutrition resulting from heat exposure

Assuming a causal association, we estimated the attributable burden of hospitalization for undernutrition due to heat exposure. In each city, we applied the following equation [27,28]:

$${AC}_{i}=C_{i}\times ({RR}_{i}-1)/{RR}_{i}$$ (2)

where *i* represents 1 day; *C*_*i*_ is the city-specific 8-day average (from day *i* to day *i* + 7) number of undernutrition hospitalization cases; RR_*i*_ is the city-specific cumulative relative risk during lag days 0–7 associated with the increase in temperature above the city-specific reference temperature for day *i*. The city-specific minimum daily mean temperature in the hot season was used as the reference temperature for that city, as our preliminary analyses had shown that the association between temperature and undernutrition hospitalization was linear. RR_*i*_ was calculated by the following equation:

RRi=ORevery1°Cincrease^(Ti−Tref) (3)

where *T*_*i*_ is the city-specific daily mean temperature on day *i*; *T*_ref_ is the city-specific reference temperature; and OR_every 1°C increase_ is the region-specific effect estimate from case-crossover analyses in the region where the city is located [27,28]. The 95% CI of AC_*i*_ was calculated using the same equations, only replacing OR_every 1°C increase_ with its 95% CI. We generated the total attributable cases (AC) and their 95% CIs by summing the AC_*i*_ values and their 95% CIs for all days in all included cities. The corresponding attributable fractions (AFs) and their 95% CIs were calculated by dividing the total AC values and their 95% CIs by total undernutrition hospitalization cases. Region-year-specific AF was calculated by dividing region-year-specific AC by region-year-specific total undernutrition hospitalization cases.

We used linear mixed effect regression models to test for a secular linear trend of the AFs and mean temperature during the hot season. In these regression models, region-year-specific AF (or average daily mean temperature during the hot season) was the dependent variable, and year (continuous variable) was the independent variable, adjusting for the random effect of region. We also fitted the relationship between region-year-specific AFs and mean temperatures (average daily mean temperature during the hot season) using a linear mixed effect model, only adjusting for the random effect of region.

We used R software (version 3.3.2) to perform all data analyses. The packages *dlnm*, *survival*, and *mvmeta* were used to fit a distributed lag linear or nonlinear model, conditional logistic regression, and meta-regression, respectively [24]. The package *nlme* was used to fit linear mixed effect models. A 2-sided *p-*value less than 0.05 was considered statistically significant.

## Results

The median daily mean temperature was 25.7°C (inter quartile range [IQR]: 23.9–27.5°C) during the hot season in all cities included, ranging from 28.0°C (IQR: 27.0–28.8°C) in the North region to 23.9°C (IQR: 22.1–25.5°C) in the South region during 2000−2015. Overall, there was a total of 238,320 (44.5% female) hospitalizations for undernutrition, with a median patient age of 57.9 years (IQR: 34.9–75.1 years). Among hospitalizations with a specific primary diagnosis, severe PEM was most frequent (accounted for 52.3%; 44.6% female; median age 50.7 years), followed by moderate PEM (32.3%; 46.1% female; median age 55.2 years). However, a large proportion of the included hospitalizations were associated with unspecified PEM (63.1%; 44.1% female; median age 60.2 years) (Table 1).

**Table 1:** Summary of hospitalizations for undernutrition, and daily mean temperature by region, in 1,814 Brazilian cities during the 2000−2015 hot seasons.

Characteristic	Number of cities	Population coverage (%)	Cases of different types of undernutrition						Daily mean temperature (°C), median (IQR)
			Severe PEM	Moderate PEM	Mild PEM	Retarded development following PEM	Unspecified PEM	Total	
**Total**	1,814	78.4	46,100	28,433	12,266	1,202	150,319	238,320	25.7 (23.9–27.5)
**Region**									
North	28	26.3	610	289	178	7	1,676	2,760	26.5 (25.1–27.9)
Northeast	662	78.0	13,545	10,852	3,191	427	58,513	86,528	28.0 (27.0–28.8)
Central West	128	80.7	2,673	1,389	563	126	7,288	12,039	27.4 (26.1–28.8)
Southeast	622	87.0	21,754	10,790	6,321	482	64,341	103,688	23.9 (22.1–25.5)
South	374	83.2	7,518	5,113	2,013	160	18,501	33,305	24.6 (23.2–25.9)
**Female, percent**	—	—	44.6	46.1	43.7	48.6	44.1	44.5	—
**Age, median (IQR)**	—	—	50.7 (4.1–72.1)	55.2 (23.5–74.3)	57.9 (37.7–75.0)	46.0 (1.1–71.3)	60.2 (40.5–75.8)	57.9 (34.9–75.1)	—

IQR, interquartile range; PEM, protein–energy malnutrition.

### Association between temperature and hospitalization for undernutrition

**Fig 2:**
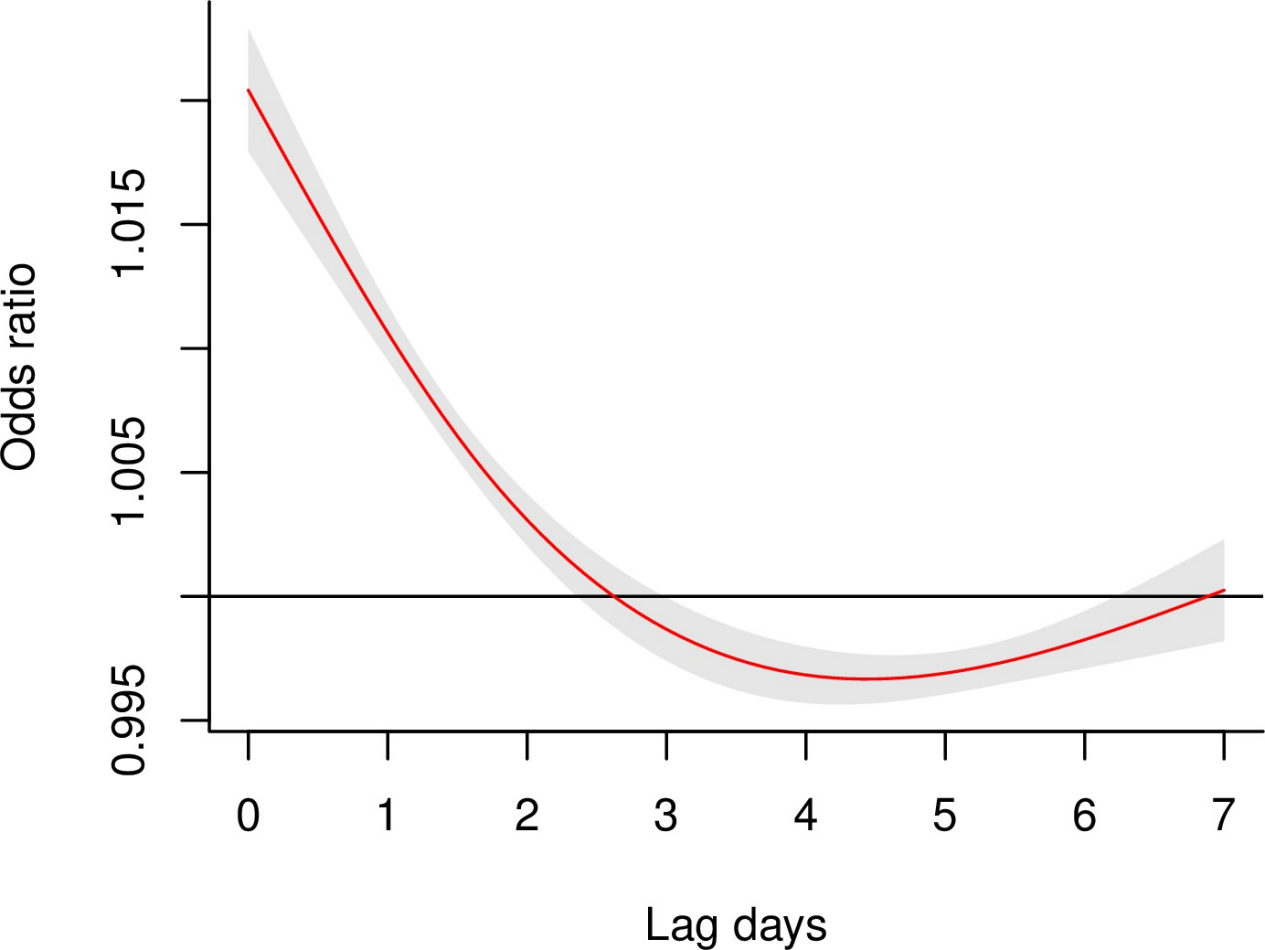
The association between heat exposure (every 1°C increase in daily mean temperature during the hot season) and hospitalization for undernutrition across 0–7 lag days. The shaded area represents the 95% confidence interval of the odds ratio. The model adjusted for potential changes in hospital use during public holidays, and also controlled for all potential time-constant confounders (e.g., age, sex, income, and lifestyle) and time-dependent confounders (e.g., temporal trend and day of the week) by its design.

The association between temperature and hospitalization for undernutrition was linear (S1 Fig). The effects of heat exposure on hospitalization were acute, but followed by temporal displacement, or harvesting effect, until the third day. In other words, some undernutrition hospitalizations that might have been expected on lag days 3–6 happened in advance, on lag days 0–2 due to heat exposure on lag day 0 (Fig 2).

**Fig 3:**
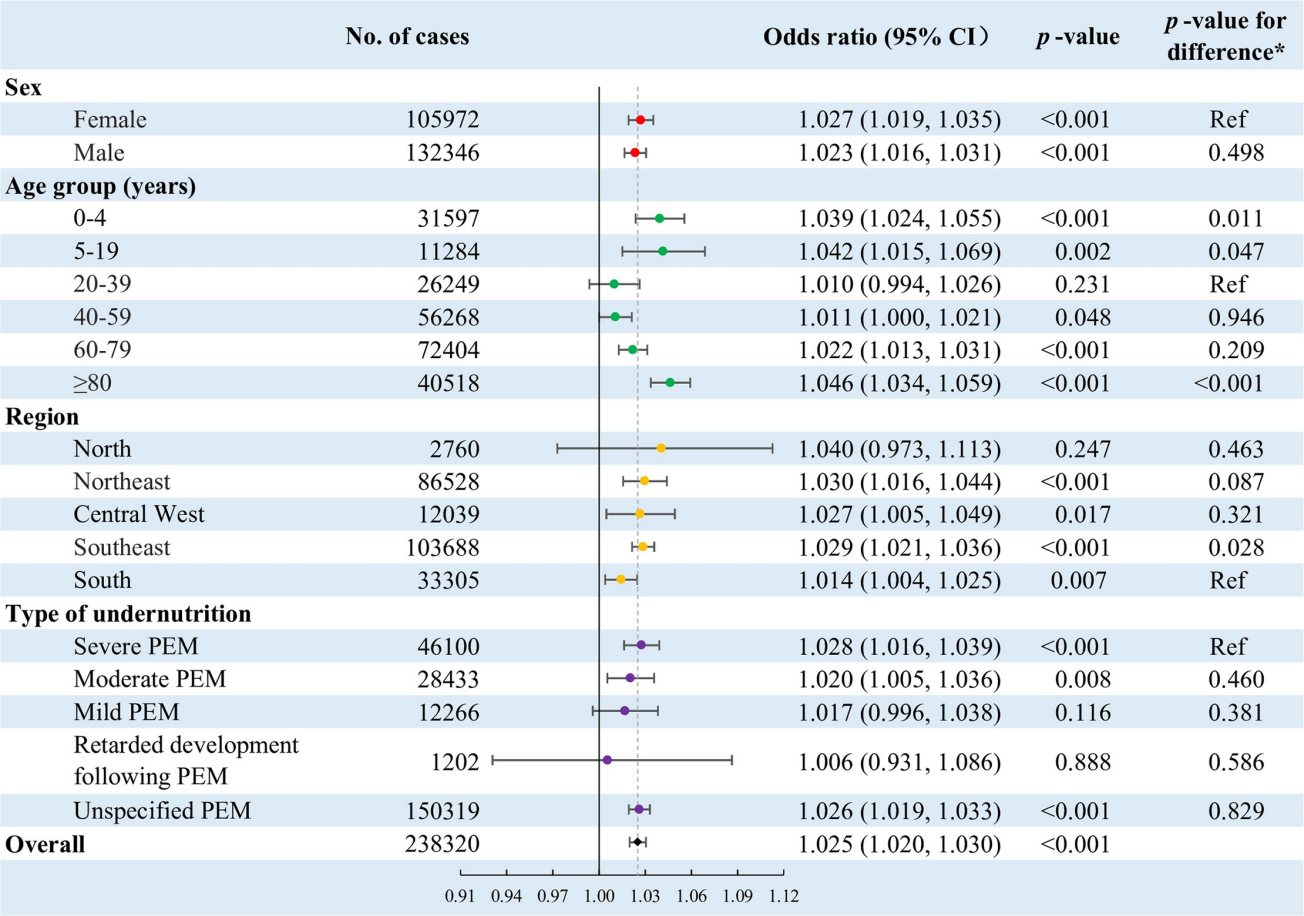
Association between heat exposure (every 1°C increase in daily mean temperature during the hot season) and hospitalization for undernutrition over 0–7 lag days. *p-Values for testing the difference between subgroups, estimated by meta-regression. The overall estimate is the effect estimate based on the complete sample, not the pooled result of any subgroup analyses by meta-analysis. The vertical solid line represents the reference line for odds ratio = 1, helping to compare the effect estimates with the null hypothesis. The vertical dashed line represents the reference line for the odds ratio equal to the overall effect estimate, helping to compare the subgroup effect estimates with the overall effect estimate. The horizontal error bars represent 95% CIs. The model adjusted for potential changes in hospital use during public holidays, and also controlled for all potential time-constant confounders (e.g., age, sex, income, and lifestyle) and time-dependent confounders (e.g., temporal trend and day of the week) by its design. CI, confidence interval; PEM, protein–energy malnutrition.

Fig 3 shows that every 1°C increase in daily mean temperature was associated with a 2.5% (OR 1.025, 95% CI 1.020–1.030, *p <* 0.001) greater risk of hospitalization for undernutrition for lag days 0–7 at the national level. The estimate of the effect was as strong in males as in females. However, the association was significantly modified by age. The effect size increased across successive age groups 40 years and older, and was maximal in those aged ≥80 years (OR 1.046, 95% CI 1.034–1.059, *p <* 0.001). Children and adolescents aged 0–4 years (OR 1.039, 95% CI 1.024–1.055, *p <* 0.001) and 5–19 years (OR 1.042, 95% CI 1.015–1.069, *p =* 0.002) also showed a stronger effect size compared to those aged 20–39 years (Fig 3).

The strength of the association between temperature and hospitalization for undernutrition showed no significant variation (*p*-values for the difference all >0.05) between the regions North, Northeast, Central West, and Southeast. However, it seemed that the association was weaker in the South region compared to the other regions. There was no strong evidence that the association varied by type of undernutrition (*p*-values for the difference all >0.05). However, there was a pattern that the effect size strengthened with the severity of undernutrition. The OR (95% CI) increased from 1.017 (0.996–1.038, *p =* 0.116) for mild PEM to 1.028 (1.016–1.039, *p <* 0.001) for severe PEM. Hospitalization due to retarded development following PEM revealed a very weak and nonsignificant association with heat exposure, although this is likely related to the limited sample size (Fig 3).

### Attributable burden of hospitalization for undernutrition due to heat exposure

**Fig 4:**
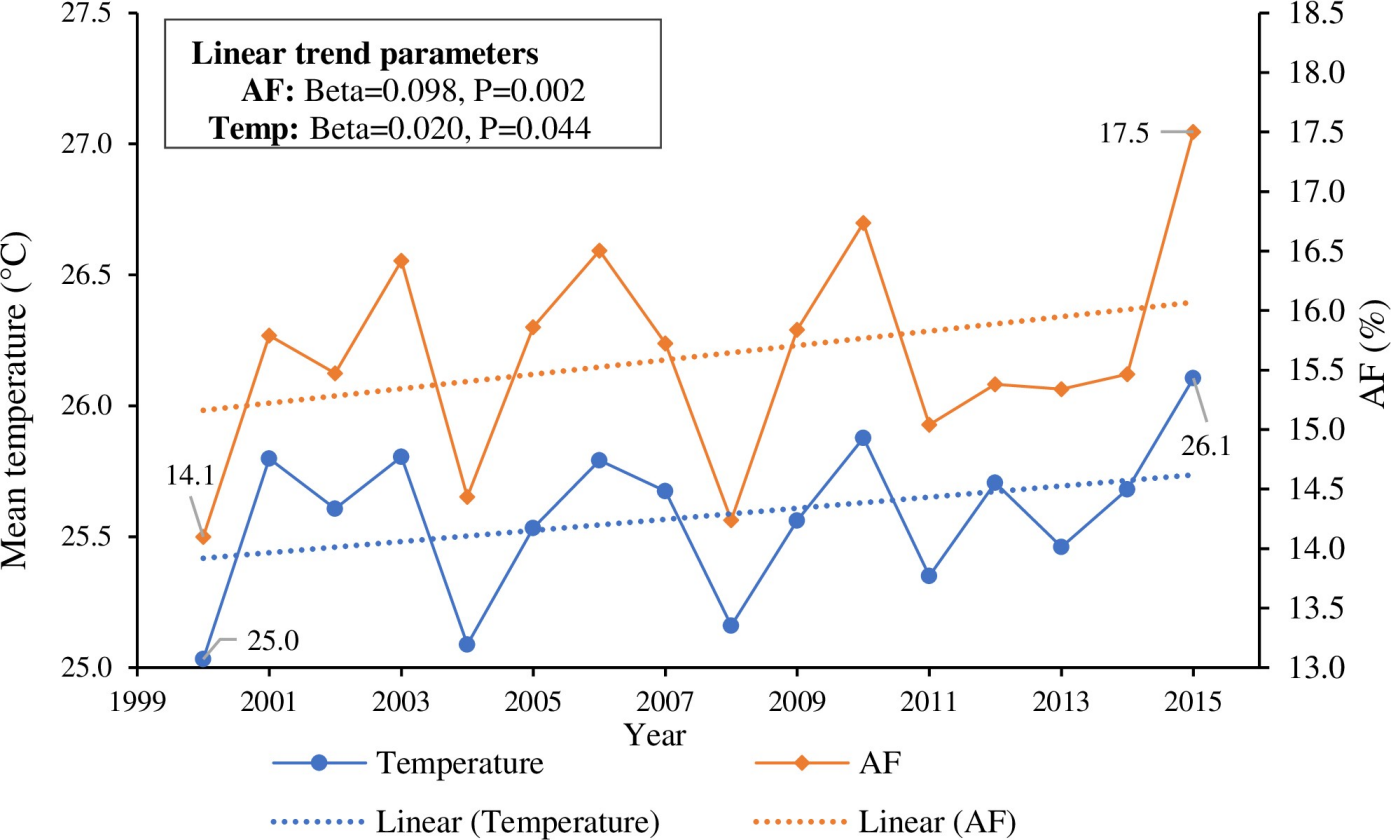
The long-term trends of daily mean temperature and fraction of hospitalizations for undernutrition attributable to heat exposure during the hot season from 2000 to 2015 in Brazil. AF, attributable fraction.

Overall, 15.6% (95% CI 9.0%–21.4%) of undernutrition hospitalizations during the hot season (equivalent to 37,129 [95% CI 21,511–51,032] hospitalization cases) could be attributed to heat exposure during the study period. This fraction was especially high in the elderly (aged ≥80 years) and children and adolescents (aged 0–19 years), with over one-quarter of admissions for undernutrition related to heat exposure (Table 2). Over the 16-year study period, the AF increased from 14.1% to 17.5% (*p* for trend = 0.002), paralleling a 1.1°C increase in daily mean temperature (*p* for trend = 0.044) (Fig 4).With every 1°C increase in region-year-specific average daily mean temperature during the hot season, the region-year-specific AF was estimated to increase by 2.5 percentage points on average (*p* < 0.001) (S2 Fig).

**Table 2:** The fraction and number of cases of hospitalization for undernutrition attributable to heat exposure during the hot season from 2000 to 2015 in Brazil.

Subgroup	Attributable fraction (95% CI), percent	Number of attributable cases (95% CI)
**Sex**		
Female	16.7 (6.8, 25.0)	17,666 (7,161, 26,521)
Male	14.7 (5.5, 22.5)	19,432 (7,283, 29,784)
**Age group (years)**		
0–19	25.1 (9.6, 37.1)	10,772 (4,129, 15,904)
20–39	2.8 (−21.4, 20.6)	740 (−5,607, 5,418)
40–59	6.8 (−11.2, 19.8)	3,844 (−6,317, 11,118)
60–79	12.6 (−0.3, 23.0)	9,142 (−244, 16,669)
≥80	26.8 (11.7, 38.1)	10,879 (4,755, 15,429)
**Region**		
North	22.2 (−19.5, 48.2)	612 (−538, 1,329)
Northeast	13.5 (7.4, 19.2)	11,705 (6,409, 16,619)
Central West	21.3 (4.2, 35.0)	2,560 (510, 4,219)
Southeast	17.3 (13.4, 21.1)	17,968 (13,871, 21,860)
South	12.9 (3.8, 21.0)	4,284 (1,258, 7,005)
**Overall**	15.6 (9.0, 21.4)	37,129 (21,511, 51,032)

CI, confidence interval.

Our results were robust to changing the maximum lag of daily mean temperature and df of lag days (S2 Table). Effect estimates from models adjusting for relative humidity in a subsample of 193 cities had no statistically significant difference from effect estimates from the primary model (S3 Table). The results changed slightly when adding more months to the hot season (S4 Table). Every 1°C increase in daily mean temperature was associated with a smaller increase in hospitalization for undernutrition in the cold months (OR 1.011) and the moderate months (OR 1.017) compared to the hot season (OR 1.025; *p*-value for difference < 0.05) (S5 Table).

## Discussion

This is the first study to our knowledge to evaluate the association between heat exposure and risk of hospitalization for undernutrition. Our findings indicate that in the Brazilian population, short-term heat exposure during the hot season was significantly associated with a greater risk of undernutrition hospitalization. Overall, assuming a cause–effect relation, about 15% of hospitalizations for undernutrition could be attributed to heat exposure. The nature of the relationship was consistent in women and men, but was stronger among the elderly and children and adolescents than among other age groups.

Our previous study reported that every 5°C increase in daily mean temperature during the 2000–2015 hot seasons in Brazil was associated with a 4.0% increase in all-cause hospitalization over lag 0–7 days, reflecting a 14% increase in infectious and parasitic hospitalizations, an 11% increase in endocrine and metabolic hospitalizations, and a less than 8% increase in all other types of hospitalization (e.g., about a 3% increase in respiratory hospitalizations) [13]. Therefore, it seems that hospitalization for undernutrition (13% increase associated with every 5°C increase in temperature) is more sensitive to heat exposure than hospitalization for other conditions, except for infectious and parasitic hospitalization.

The underlying pathways behind the observed association between heat exposure and increased risk of hospitalization for undernutrition are not very well understood. We speculate that there are several possible pathways based on the current knowledge. First, hot weather may reduce undernourished people’s food intake by reducing their appetites, provoking more alcohol consumption, or making them unable or lacking motivation to shop and cook [29]. Lack of food intake would exacerbate any undernutrition and may finally result in hospital admission. Second, hot weather could potentially worsen undernourished people’s already impaired digestion and absorption by increasing gastrointestinal morbidity, e.g., gastroenteritis [30]. This may also aggravate their undernutrition condition. Third, individuals suffering from undernutrition have impaired thermoregulation [29,31]. Peripheral nutritional neuropathy may impair both peripheral thermal sensors and efferent responses to temperature changes [32]. PEM has also been shown to affect central thermoregulatory structures, inducing significant abnormalities in central core temperature circadian rhythm [33]. When exposed to high temperatures, undernourished people are more likely to develop fluid and electrolyte disturbances than well-nourished populations due to a lack of capacity to dissipate heat. Finally, undernourished people generally come from low socioeconomic communities, which means they also lack the ability to mitigate heat exposure by using strategies such as staying indoors with an air conditioner [34].

The greater susceptibility of elderly people and children and adolescents to heat exposure is consistent with our previous finding [13]. A possible explanation may be the immature or impaired thermoregulation in these 2 age groups [13]. It is anticipated that the proportion of people aged 65 years or above in Brazil will rise to about 35% by 2040 [35]. The rapid population aging in Brazil is likely to increase the burden of heat-related undernutrition morbidity even further by producing a more vulnerable population. The observed regional variation in the association between heat and hospitalization for undernutrition might reflect contributions by many factors, such as climatic characteristics, population structure, socioeconomic level, and occupation types (outdoor jobs versus indoor jobs) [12,13,36]. For example, the South region has the highest literacy rate compared to the other regions in Brazil [37], which might explain its weaker association between heat and hospitalization for undernutrition. However, more studies are wanted to reveal the contributing factors for regional variation in heat vulnerability.

Climate change is one of the biggest threats to the reduction of hunger and undernutrition, especially in LMICs [5–7]. It has been estimated that climate change will reduce global food availability by 3.2% and thus cause about 30,000 underweight-related deaths by 2050 [38]. However, this may actually underestimate the real effect of climate change on future undernutrition-related morbidity and mortality, because it overlooks the direct and short-term effects of temperature rise. We estimated that over 15% of undernutrition hospitalizations could have been attributable to heat exposure in Brazil during the study period. It is plausible to speculate that climate changes could not only increase the rate of undernutrition in the most affected areas of the globe, but also, at same time, impair individuals’ capacity to adapt to projected rises in temperature.

The inadequate climate change mitigation response is putting the world on a high-end emissions trajectory that will result in a 2.6–4.8°C temperature rise by the end of this century [7]. Based on our finding, such a magnitude of temperature rise would make the AF of undernutrition hospitalization due to heat exposure rise by 6.6–12.1 percentage points (temperature rise multiplied by 2.5; see S2 Fig), assuming other factors remained unchanged. Therefore, short-term heat exposure will be an increasingly important driver of undernutrition morbidity in the future. Thus, global strategies addressing the syndemic of climate change and undernutrition should not only focus on food security [5], but also pay attention to dealing with heat exposure.

The present study has several strengths. First, to the best of our knowledge, this is the first study that has evaluated the association between temperature and hospitalization for undernutrition. The results are expected to be statistically robust and stable because of our large sample size. Second, Brazil is a large country with significant diversity in temperatures; thus, our results, especially our region-specific results, may also apply to other countries with similar climates. Third, our study represents Brazil well both geographically and temporally via access to a national dataset covering nearly 80% of the Brazilian population and spanning 16 years. Evidence from one of the biggest middle-income countries may also have implications for other large middle-income nations (e.g., China and India).

However, several limitations of this study should also be acknowledged. First, the classification of undernutrition types was possibly not accurate enough, given the large proportion of hospitalized cases due to unspecified undernutrition. Fortunately, the effect estimates varied little among different types of undernutrition; thus, the bias due to outcome misclassification should be minimal. Second, we could only get access to city-level temperature data instead of individual-level data. This measurement error tends to be independent of the true exposure level, and to be nondifferential (random). Therefore, our analysis may underestimate the temperature–hospitalization association [39,40]. Finally, we were unable to adjust for relative humidity and air pollution in the main model due to limited access to relevant data. However, our sensitivity analyses indicated that adjustment for relative humidity in the dataset of 193 cities that had humidity data had no significant influence on the results.

Some researchers have argued that air pollution should not be adjusted for when evaluating the health effect of ambient temperature [41]. The main reason is that daily ambient temperature influences air pollution levels rather than the converse, so air pollution is more likely to be a mediator rather than a true confounder between temperature and health. Moreover, the association between temperature and health often changes only minimally after adjustment for humidity or air pollutants, as suggested by previous studies [42,43].

In conclusion, heat exposure is associated with increased risk of hospitalization for undernutrition, especially among the elderly, children, and adolescents. These findings highlight the short-term and direct impacts of global warming on undernutrition morbidity. This kind of impact is anticipated to be increasingly important in the future, because of global warming.

## Supporting information

S1 RECORD Checklist The RECORD statement checklist of items, extended from the STROBE statement, that should be reported in observational studies using routinely collected health data. (DOCX)

S1 Fig The linearity of the relationship between daily mean temperature and hospitalization for undernutrition across lag 0–7 days during the hot season at the national level, modeled by a distributed lag nonlinear model. The odds ratio is the odds ratio of hospitalization for undernutrition at a given temperature compared to the reference temperature (the temperature when log[odds ratio] = 0). We selected as the reference temperature the median temperature during the study period (25.7°C). The shaded area represents the 95% confidence interval of the odds ratio.
(TIF)

S2 Fig The association between region-year-specific attributable fraction (AF) and mean temperature (region-year-specific average daily mean temperature during the hot season) in Brazil in 2000−2015. AF is the AF of undernutrition hospitalization due to heat exposure. The shaded area represents the 95% confidence interval of the solid line.
(TIF)

S1 Table Example data for conditional logistic regression. (DOCX)

S2 Table Results of sensitivity analyses changing maximum lag days of daily mean temperature and df of lag days. (DOCX)

S3 Table Results of sensitivity analysis adjusting for relative humidity using data from 193 cities. (DOCX)

S4 Table Results of sensitivity analyses including more months in the hot season. (DOCX)

S5 Table The association between temperature and hospitalization for undernutrition in the hot season, moderate months, and cold months. (DOCX)

S1 Text Prospective analysis plan and modifications following comments from editors and reviewers. (DOCX)

## Abbreviations

AF: attributable fraction
CI: confidence interval
BUHS: Brazilian Unified Health System
df: degrees of freedom
IQR: interquartile range
LMICs: low- and middle-income countries
OR: odds ratio
PEM: protein–energy malnutrition
