# Supplementary material for: The association between heat exposure and hospitalization for undernutrition in Brazil during 2000−2015: A nationwide case-crossover study
Source: PLoS Med. 2019 Oct 29;16(10):e1002950. doi: 10.1371/journal.pmed.1002950 (PMC6818759; doi:10.1371/journal.pmed.1002950)
Supplement: S1 RECORD Checklist — (DOCX) [file pmed.1002950.s001.docx]

**The RECORD statement – checklist of items, extended from the STROBE statement, that should be reported in observational studies using routinely collected health data.**

|  | **Item No.** | **STROBE items** | **Location in manuscript where items are reported** | **RECORD items** | **Location in manuscript where items are reported** |
| --- | --- | --- | --- | --- | --- |
| **Title and abstract** | | | | | |
|  | 1 | (a) Indicate the study’s design with a commonly used term in the title or the abstract (b) Provide in the abstract an informative and balanced summary of what was done and what was found | (a) In Title: “case-crossover study”; in abstract, “time-stratified case-crossover design”.  (b)In Abstract: Methods and Findings section. | RECORD 1.1: The type of data used should be specified in the title or abstract. When possible, the name of the databases used should be included.  RECORD 1.2: If applicable, the geographic region and timeframe within which the study took place should be reported in the title or abstract.  RECORD 1.3: If linkage between databases was conducted for the study, this should be clearly stated in the title or abstract. | 1.1 In Title, “hospitalization”;  1.2 In Title, “Brazil during 2000−2015”  1.3 In Abstract, “We collected hospitalization and weather data” |
| **Introduction** | | | | | |
| Background rationale | 2 | Explain the scientific background and rationale for the investigation being reported | In para 1-2 of Introduction, |  |  |
| Objectives | 3 | State specific objectives, including any prespecified hypotheses | In para 3 of Introduction |  |  |
| **Methods** | | | | | |
| Study Design | 4 | Present key elements of study design early in the paper | In the first sentence of Methods, “time-stratified case-crossover study”; Details of study design are presented in the Statistical analyses section of Methods. |  |  |
| Setting | 5 | Describe the setting, locations, and relevant dates, including periods of recruitment, exposure, follow-up, and data collection | In the Data collection section of Methods. |  |  |
| Participants | 6 | *(a) Cohort study* - Give the | NA | RECORD 6.1: The methods of study population selection (such as codes or algorithms used to identify subjects) should be listed in detail. If this is not possible, an explanation should be provided. | 6.1 In the para 1 of Data collection in  Methods: “We extracted hospitalization data with ICD-10 codes (E40-E46) for undernutrition” |

|  |  | eligibility criteria, and the sources and methods of selection of participants. Describe methods of follow-up  *Case-control study* - Give the eligibility criteria, and the sources and methods of case ascertainment and control selection. Give the rationale for the choice of cases and controls *Cross-sectional study* - Give the eligibility criteria, and the sources and methods of selection of participants  *(b) Cohort study* - For matched studies, give matching criteria and number of exposed and unexposed  *Case-control study* - For matched studies, give matching criteria and the number of controls per case |  | RECORD 6.2: Any validation studies of the codes or algorithms used to select the population should be referenced. If validation was conducted for this study and not published elsewhere, detailed methods and results should be provided.  RECORD 6.3: If the study involved linkage of databases, consider use of a flow diagram or other graphical display to demonstrate the data linkage process, including the number of individuals with linked data at each stage. | 6.2 In Data collection of Methods, we provided the WHO’s web link of ICD-10 code for undernutrition.  6.3 In Data collection of Methods, para1: “We used the weather of city centre to represent each city”. “Daily weather data were linked to hospitalization cases according to city.”  Because the linkage is simple, we did not provide a flow diagram. |
| --- | --- | --- | --- | --- | --- |
| Variables | 7 | Clearly define all outcomes, exposures, predictors, potential confounders, and effect modifiers. Give diagnostic criteria, if applicable. | In Data collection of Methods: outcome, effect modifiers (sex, age, region, type of undernutrition) have been defined in para 1, exposure (temperature) and the potential confounder (relative humidity) have been described in para 2 and para 3. | RECORD 7.1: A complete list of codes and algorithms used to classify exposures, outcomes, confounders, and effect modifiers should be provided. If these cannot be reported, an explanation should be provided. | In Data collection of Methods, para1, we provided the ICD-10 codes of undernutrition outcome. In para 2, we described that the daily mean temperature was calculated as the average of daily minimum and maximum temperature. Other variable do not have specific code or algorithms |
| Data sources/ measurement | 8 | For each variable of interest, give sources of data and details of methods of assessment (measurement).  Describe comparability of assessment methods if there is more than one group | In Data collection of Methods: para 1 described the data source of outcome and effect modifiers (sex, age, region, type of undernutrition), para 2 described the source of environmental exposure data and potential confounder (relative humidity). |  |  |
| Bias | 9 | Describe any efforts to address potential sources of bias | These have been described in the sensitivity analyses in Statistical analyses of Method. |  |  |

| Study size | 10 | Explain how the study size was arrived at | In para 1 of Data collection, we described in detail why we chose the 1,814 cities. |  |  |
| --- | --- | --- | --- | --- | --- |
| Quantitative variables | 11 | Explain how quantitative variables were handled in the analyses. If applicable, describe which groupings were chosen, and why | In para 2 of Data collection, “daily mean temperature (as approximated the average of daily minimum and maximum temperatures) during hot season was used to represent heat exposure.”  The para 3 of Statistical analyses, we described how we stratified age into six groups. |  |  |
| Statistical methods | 12 | 1. Describe all statistical methods, including those used to control for confounding 2. Describe any methods used to examine subgroups and interactions 3. Explain how missing data were addressed 4. *Cohort study* - If applicable, explain how loss to follow-up was addressed   *Case-control study* - If applicable, explain how matching of cases and controls was addressed  *Cross-sectional study* - If applicable, describe analytical methods taking account of sampling strategy   1. Describe any sensitivity analyses | (a) In Statistical analyses, para 1 and para 2.  (b) In Statistical analyses, para 3.  (c) In para 1 of Data collection, we described that we only included 1,814 cities with completed record of hospitalization. In Statistical analyses, para 3, we described how we addressed missing values.  (d) NA  (e) In Sensitivity analyses section of Statistical analyses. |  |  |
| Data access and cleaning methods |  | .. |  | RECORD 12.1: Authors should describe the extent to which the investigators had access to the database population used to create the study population.  RECORD 12.2: Authors should provide information on the data cleaning methods used in the study. | 12.1 In para 1 of Data collection, “The authors have access to the full hospitalization data recorded by BNUHS during the study period.”  12.2 In para 1 of Data collection, “However, to minimize the effects of missing values, we only selected data from 1,814 cities with completed hospitalization record over the 16 years.”  In Statistical analyses, para 3, we described how we addressed missing values. |
| Linkage |  | .. |  | RECORD 12.3: State whether the study included person-level, institutional-level, or other data linkage across two or more databases. The methods of linkage and methods of linkage quality evaluation should be provided. | In the para 2 of Data collection section, “Daily weather data were linked to hospitalization cases according to city. Because the linkage was simple and straightforward, quality evaluation was not applicable. |

|  |  |  |  |  |  |
| --- | --- | --- | --- | --- | --- |
| **Results** | | | | | |
| Participants | 13 | 1. Report the numbers of individuals at each stage of the study (*e.g.*, numbers potentially eligible, examined for eligibility, confirmed eligible, included in the study, completing follow-up, and analysed) 2. Give reasons for non- participation at each stage. 3. Consider use of a flow diagram | (a) In para 1 and table 1 of the Results.  (b) NA  (c) Because the selection process was simple, we did not use a diagram. | RECORD 13.1: Describe in detail the selection of the persons included in the study (*i.e.,* study population selection) including filtering based on data quality, data availability and linkage. The selection of included persons can be described in the text and/or by means of the study flow diagram. | This information has been described in detail in the Methods. Because the selection process was simple, we did not describe it in the Results. |
| Descriptive data | 14 | 1. Give characteristics of study participants (*e.g.*, demographic, clinical, social) and information on exposures and potential confounders 2. Indicate the number of participants with missing data for each variable of interest 3. *Cohort study* - summarise follow-up time (*e.g.*, average and total amount) | (a) In para 1 and table 1 of the Results.  (b) The issue of missing value has been described in para 3 of Statistical analyses section. Because there were only two cases with missing sex, we did not report missing value in Results section.  (c) NA |  |  |
| Outcome data | 15 | *Cohort study* - Report numbers of outcome events or summary measures over time  *Case-control study* - Report numbers in each exposure category, or summary measures of exposure  *Cross-sectional study* - Report numbers of outcome events or | In para 1 and table 1 of the Results. |  |  |

|  |  | summary measures |  |  |  |
| --- | --- | --- | --- | --- | --- |
| Main results | 16 | 1. Give unadjusted estimates and, if applicable, confounder- adjusted estimates and their precision (e.g., 95% confidence interval). Make clear which confounders were adjusted for and why they were included 2. Report category boundaries when continuous variables were categorized 3. If relevant, consider translating estimates of relative risk into absolute risk for a meaningful time period | (a) In Fig 2 and Fig 3, we added some notes to clarify which confounders were adjusted for. We provided 95%CI in Fig 2, Fig 3, Table 2, S1 Table, S2 Table, S1 Fig, and S2 Fig. P-values were also reported in Fig 3, S1 Table and S2 Table, where applicable.  We did not give unadjusted estimates because most confounders has been controlled automatically by the case-crossover design, and adjusting for public holiday by regression model only have minimal effect on the results.  (b) In Fig 3 and Table 2, the boundaries age groups were clearly displayed.  (c) In Table 2, we reported the attributable cases (95%CI), which could serve as an measure of absolute risk. |  |  |
| Other analyses | 17 | Report other analyses done—e.g., analyses of subgroups and interactions, and sensitivity analyses | In Statistical analyses, we describe the subgroup analyses in the last paragraph of the section of Assessing the temperature-hospitalization association. We describes how to compare subgroup difference, which is similar to interactions.  Then in the Sensitivity analyses section, we described several sensitivity analyses, |  |  |
| **Discussion** | | | | | |
| Key results | 18 | Summarise key results with reference to study objectives | In para 1 of Discussion. |  |  |
| Limitations | 19 | Discuss limitations of the study, taking into account sources of potential bias or imprecision.  Discuss both direction and magnitude of any potential bias | In para 8 and para 9 of the Discussion. We discussed three main limitations of the present study. | RECORD 19.1: Discuss the implications of using data that were not created or collected to answer the specific research question(s). Include discussion of misclassification bias, unmeasured confounding, missing data, and changing eligibility over time, as they pertain to the study being reported. | In para 8 and para 9 of the Discussion. We discussed the misclassification of outcome, the measurement error of temperature, the potential unmeasured confounding (relative humidity and air pollution). |
| Interpretation | 20 | Give a cautious overall interpretation of results considering objectives, limitations, multiplicity of analyses, results from similar studies, and other relevant evidence | In the last para of Discussion. |  |  |

| Generalisability | 21 | Discuss the generalisability (external validity) of the study results | In para 7 of Discussion, “Brazil is a large country with significant diversity in temperatures, thus our results especially regional-specific results may also apply to other countries with similar climate.” “Evidence from one of the biggest  middle-income countries may also provide implications for other large middle-income nations (e.g. China, India).” |  |  |
| --- | --- | --- | --- | --- | --- |
| **Other Information** | | | | | |
| Funding | 22 | Give the source of funding and the role of the funders for the present study and, if applicable, for the original study on which the present article is based | In Funding section |  |  |
| Accessibility of protocol, raw data, and programming code |  | .. | Analyses protocol has been described in Statistical methods and S2 Text. The accessibility of raw data and programing code has been described in Data availability statement. | RECORD 22.1: Authors should provide information on how to access any supplemental information such as the study protocol, raw data, or programming code. | Analyses protocol has been described in Statistical methods and S2 Text. The accessibility of raw data and programing code has been described in Data availability statement. We also provided a sample R code for case-crossover analyses based on a simulated dataset. |

*Reference: Benchimol EI, Smeeth L, Guttmann A, Harron K, Moher D, Petersen I, et al. (2015) The REporting of studies Conducted using Observational Routinely-collected health Data (RECORD) Statement. PLoS Med 12(10): e1001885. https://doi.org/10.1371/journal.pmed.1001885

*Checklist is protected under Creative Commons Attribution ([CC BY](http://creativecommons.org/licenses/by/4.0/)) license.
