# Supplementary figures and images for: The association between heat exposure and hospitalization for undernutrition in Brazil during 2000−2015: A nationwide case-crossover study
Source: PLoS Med. 2019 Oct 29;16(10):e1002950. doi: 10.1371/journal.pmed.1002950 (PMC6818759; doi:10.1371/journal.pmed.1002950)

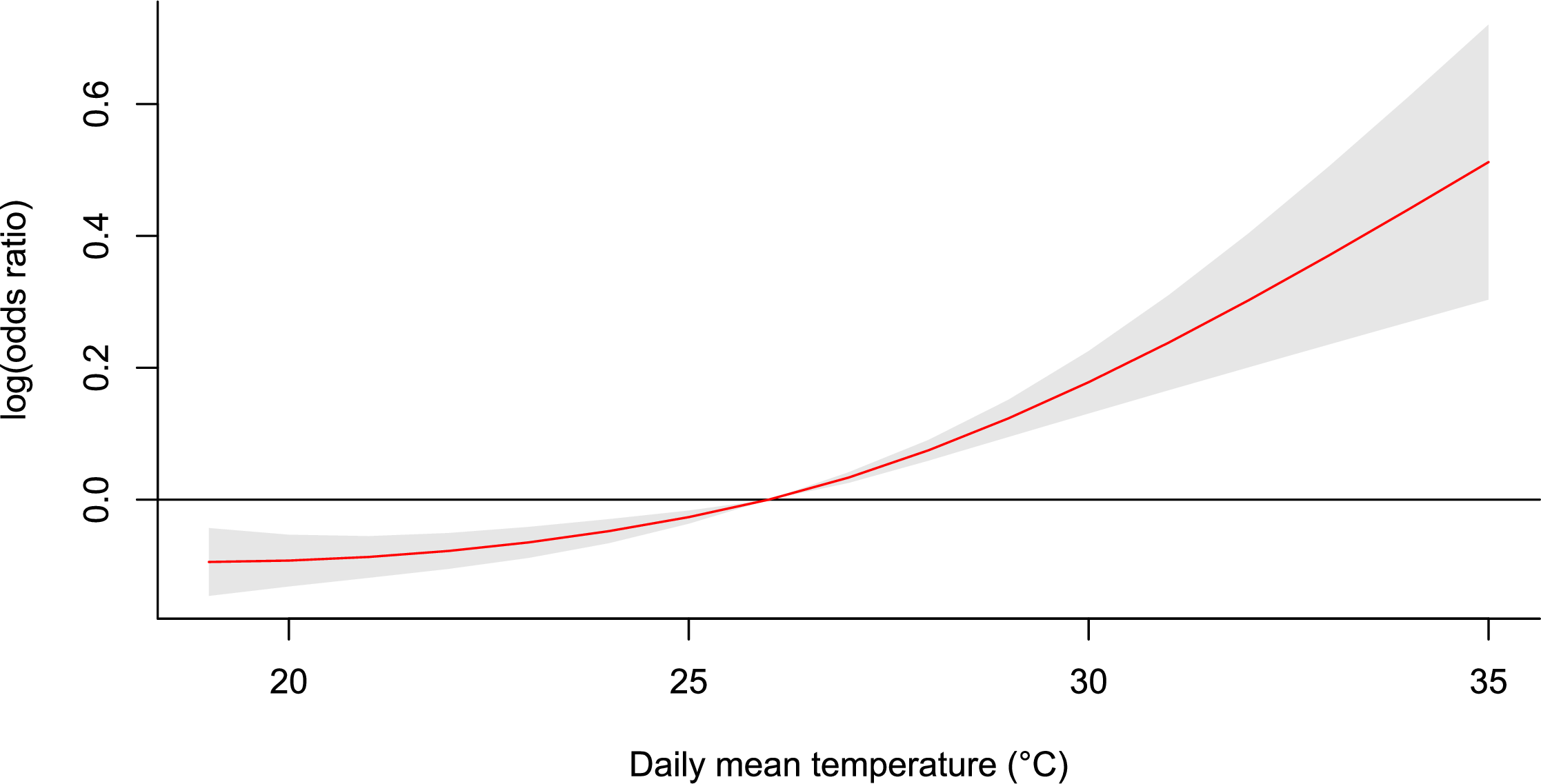

Supplement: S1 Fig — The odds ratio is the odds ratio of hospitalization for undernutrition at a given temperature compared to the reference temperature (the temperature when log[odds ratio] = 0). We selected as the reference temperature the median temperature during the study period (25.7°C). The shaded area represents the 95% confidence interval of the odds ratio. (TIF) [file pmed.1002950.s002.tif]

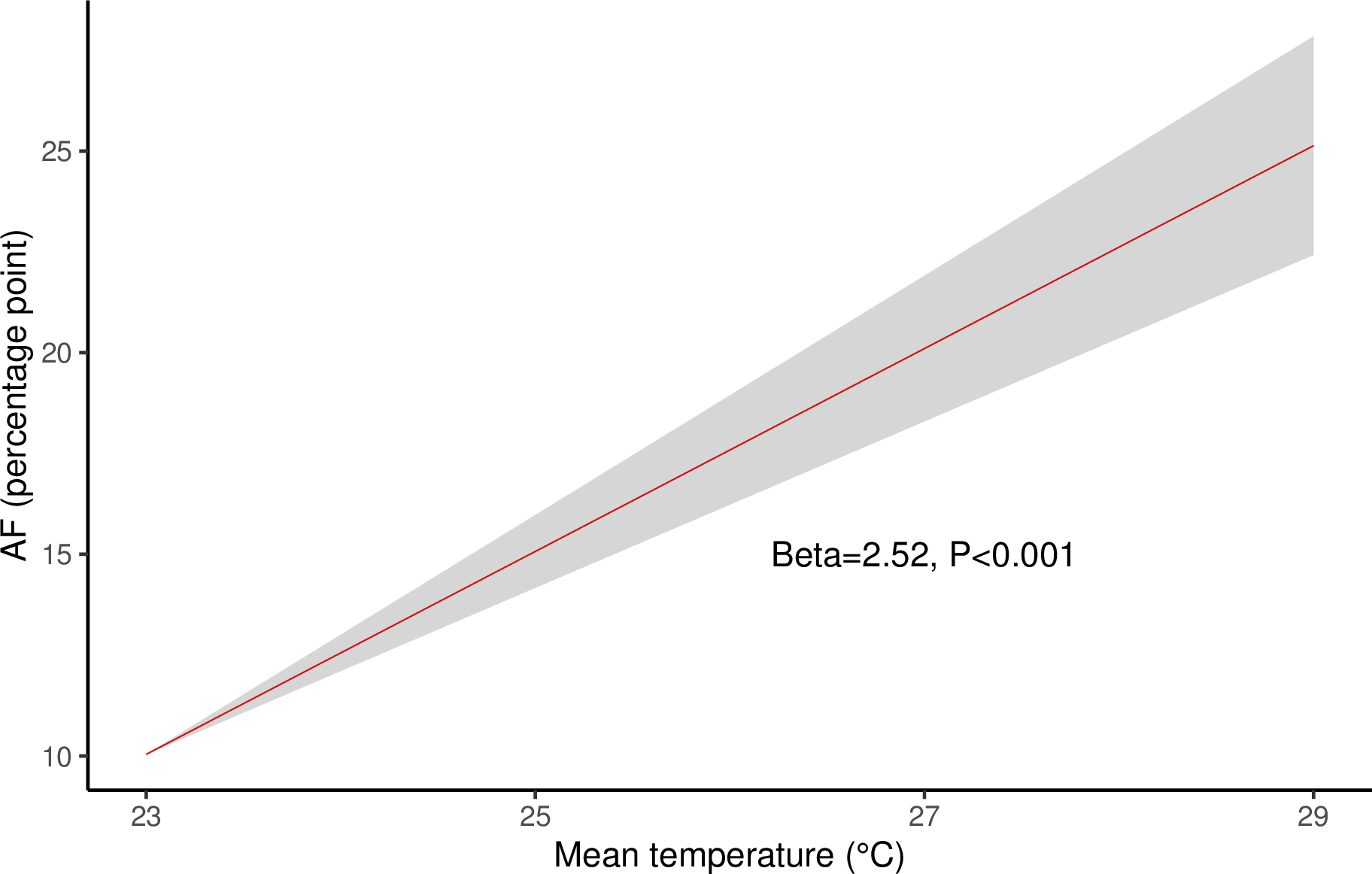

Supplement: S2 Fig — AF is the AF of undernutrition hospitalization due to heat exposure. The shaded area represents the 95% confidence interval of the solid line. (TIF) [file pmed.1002950.s003.tif]
