# Supplementary material for: The association between heat exposure and hospitalization for undernutrition in Brazil during 2000−2015: A nationwide case-crossover study
Source: PLoS Med. 2019 Oct 29;16(10):e1002950. doi: 10.1371/journal.pmed.1002950 (PMC6818759; doi:10.1371/journal.pmed.1002950)
Supplement: S1 Table — (DOCX) [file pmed.1002950.s004.docx]

**S1 Table.** Example data for conditional logistic regression.

| Stratum | date | dow | case | lag0 | lag1 | lag2 | lag3 | lag4 | lag5 | lag6 | lag7 | holidays |
| --- | --- | --- | --- | --- | --- | --- | --- | --- | --- | --- | --- | --- |
| 1 | 3/08/2000 | Thursday | 1 | 27.3 | 27.6 | 26.8 | 27.0 | 27.1 | 27.0 | 27.5 | 26.7 | 0 |
| 1 | 10/08/2000 | Thursday | 0 | 28.0 | 27.8 | 27.8 | 27.8 | 27.9 | 27.8 | 27.4 | 27.3 | 0 |
| 1 | 17/08/2000 | Thursday | 0 | 27.0 | 26.9 | 27.8 | 27.6 | 28.0 | 28.4 | 27.6 | 28.0 | 0 |
| 1 | 24/08/2000 | Thursday | 0 | 27.9 | 28.5 | 28.9 | 28.2 | 28.0 | 27.2 | 27.5 | 27.0 | 0 |
| 2 | 17/03/2000 | Friday | 1 | 26.5 | 26.0 | 22.8 | 22.6 | 25.8 | 25.6 | 25.3 | 25.4 | 0 |
| 2 | 24/03/2000 | Friday | 0 | 27.2 | 26.8 | 26.7 | 26.3 | 25.6 | 26.1 | 26.4 | 26.5 | 0 |
| 2 | 31/03/2000 | Friday | 0 | 25.9 | 24.2 | 23.1 | 23.1 | 27.0 | 26.9 | 26.6 | 27.2 | 0 |
| 2 | 10/03/2000 | Friday | 0 | 25.4 | 24.2 | 25.6 | 27.0 | 26.0 | 25.8 | 25.9 | 27.0 | 0 |
| 2 | 3/03/2000 | Friday | 0 | 27.0 | 27.1 | 27.2 | 26.4 | 25.7 | 28.1 | 28.0 | 28.2 | 0 |
| 3 | 11/12/2000 | Monday | 1 | 31.6 | 30.5 | 31.0 | 28.4 | 27.8 | 26.6 | 27.0 | 26.6 | 0 |
| 3 | 18/12/2000 | Monday | 0 | 25.9 | 23.5 | 28.6 | 29.0 | 26.2 | 28.8 | 30.6 | 31.6 | 0 |
| 3 | 25/12/2000 | Monday | 0 | 29.0 | 27.2 | 31.9 | 30.6 | 29.6 | 28.4 | 27.7 | 25.9 | 1 |
| 3 | 4/12/2000 | Monday | 0 | 26.6 | 29.4 | 28.5 | 28.9 | 28.1 | 29.3 | 30.9 | 29.5 | 0 |

Note: case is a binary independent variable indicating whether the hospitalization case arose on that day (case=1) or not (case=0); a stratum is consisted of one case (case=1) day and its three or four controls days (case=0), the total number of stratums is equal to the number of hospitalization for undernutrition; lag0 to lag7 represent the daily mean temperature in 0-7 days before the case and control days, in °C (e.g., lag3 was the daily mean temperature 3 days before the date recorded by the date variable). dow means day of week; holidays is a binary variable indicating whether the date is a public holiday (holidays=1) or not (holidays=0).
