# Supplementary material for: The association between heat exposure and hospitalization for undernutrition in Brazil during 2000−2015: A nationwide case-crossover study
Source: PLoS Med. 2019 Oct 29;16(10):e1002950. doi: 10.1371/journal.pmed.1002950 (PMC6818759; doi:10.1371/journal.pmed.1002950)
Supplement: S2 Table — (DOCX) [file pmed.1002950.s005.docx]

**S2 Table.** Results of sensitivity analyses changing maximum lag days of daily mean temperature and df of lag days.

| Model | Odds ratio (95% CI) | *p-value* | *p*-value for difference* |
| --- | --- | --- | --- |
| Primary | 1.025 (1.020, 1.030) | <0.001 | Ref |
| Lag 0-5 days | 1.028 (1.023, 1.032) | <0.001 | 0.476 |
| Lag 0-6 days | 1.026 (1.021, 1.031) | <0.001 | 0.880 |
| Lag 0-8 days | 1.023 (1.018, 1.029) | <0.001 | 0.677 |
| Lag 0-9 days | 1.021 (1.016, 1.027) | <0.001 | 0.372 |
| Lag 0-10 days | 1.019 (1.013, 1.026) | <0.001 | 0.172 |
| df of lag days = 4 | 1.026 (1.020, 1.031) | <0.001 | 0.892 |

Note: df=degree of freedom. Odds ratio represents the overall association between every 1°C increase in daily mean temperature over lag days and hospitalization for undernutrition. *P*-value for difference were estimated by fixed effect meta-regression with no statistical adjustment, because those models were based on the same sample.
