# Supplementary material for: The association between heat exposure and hospitalization for undernutrition in Brazil during 2000−2015: A nationwide case-crossover study
Source: PLoS Med. 2019 Oct 29;16(10):e1002950. doi: 10.1371/journal.pmed.1002950 (PMC6818759; doi:10.1371/journal.pmed.1002950)
Supplement: S3 Table — (DOCX) [file pmed.1002950.s006.docx]

**S3 Table.** Results of sensitivity analysis adjusting for relative humidity in lag0-7 days with different model using data of 193 cities.

| Model | Adjusted humidity | df for lag days | df for humidity | | OR (95%CI) | *p-value* | *p-value* for difference |
| --- | --- | --- | --- | --- | --- | --- | --- |
| Primary model | NA | NA | NA | 1.034 (1.022, 1.047) | | <0.001 | Ref |
| Adjusted 1 | moving average of lag0-7 days | NA | 3 | 1.022 (1.037, 1.037) | | 0.003 | 0.216 |
| Adjusted 2 | moving average of lag0-7 days | NA | 4 | 1.022 (1.038, 1.037) | | 0.003 | 0.220 |
| Adjusted 3 | crossbasis for lag0-7 days | 3 | 3 | 1.024 (1.045, 1.039) | | 0.002 | 0.287 |
| Adjusted 4 | crossbasis for lag0-7 days | 3 | 4 | 1.024 (1.044, 1.039) | | 0.002 | 0.284 |
| Adjusted 5 | crossbasis for lag0-7 days | 4 | 3 | 1.024 (1.045, 1.039) | | 0.002 | 0.297 |
| Adjusted 6 | crossbasis for lag0-7 days | 4 | 4 | 1.024 (1.045, 1.039) | | 0.002 | 0.294 |

Note: Odds ratio represents the association between every 1°C increase in daily mean temperature during the hot season and hospitalization for undernutrition; df=degree of freedom. We adjusted for relative humidity using 6 different models by using moving average or crossbasis with difference setting of relative humidity during lag 0-7 days. *P*-value for difference were estimated by fixed effect meta-regression with no statistical adjustment, because those models were based on the same sample.
