# Supplementary material for: The association between heat exposure and hospitalization for undernutrition in Brazil during 2000−2015: A nationwide case-crossover study
Source: PLoS Med. 2019 Oct 29;16(10):e1002950. doi: 10.1371/journal.pmed.1002950 (PMC6818759; doi:10.1371/journal.pmed.1002950)
Supplement: S4 Table — (DOCX) [file pmed.1002950.s007.docx]

| **S4 Table.** Results of sensitivity analyses including more months to the hot season | | | | | |
| --- | --- | --- | --- | --- | --- |
| Hot season definition | No. of cases | | OR (95%CI) | p-value | p-value for difference |
| 4 adjacent hotest months (primary model) | 238,320 | 1.025 (1.020, 1.030) | | <0.001 | Ref |
| 5 adjacent hotest months | 295,789 | 1.024 (1.019, 1.028) | | <0.001 | 0.692 |
| 6 adjacent hotest months | 353,583 | 1.023 (1.019, 1.027) | | <0.001 | 0.520 |

Note: Odds ratio represents the association between every 1°C increase in daily mean temperature during the hot season and hospitalization for undernutrition; *p*-value for difference were estimated by fixed effect meta-regression with no statistical adjustment, because those models were based on the similar sample.
