# Supplementary material for: The association between heat exposure and hospitalization for undernutrition in Brazil during 2000−2015: A nationwide case-crossover study
Source: PLoS Med. 2019 Oct 29;16(10):e1002950. doi: 10.1371/journal.pmed.1002950 (PMC6818759; doi:10.1371/journal.pmed.1002950)
Supplement: S5 Table — (DOCX) [file pmed.1002950.s008.docx]

| **S5 Table.** The association between temperature and hospitalization for undernutrition in hot season, moderate and cold months. | | | | |
| --- | --- | --- | --- | --- |
|  | No. of cases | OR (95%CI) | p-value | p-value for difference |
| Hot season | 238,320 | 1.025 (1.020, 1.030) | <0.001 | Ref |
| Moderate months | 228,448 | 1.017 (1.013, 1.021) | <0.001 | 0.017 |
| Cold months | 219,184 | 1.011 (1.007, 1.015) | <0.001 | <0.001 |

Note: Odds ratio represents the association between every 1°C increase in daily mean temperature and hospitalization for undernutrition; *p*-value for difference were estimated by random effect meta-regression fitted by maximum likelihood method, because those models were based on different samples (samples in different seasons). Hot season was the city-specific 4 adjacent hottest months; cold months was the city-specific 4 coldest months; the remained months were categorized as moderate months.
