# Supplementary material for: The association between heat exposure and hospitalization for undernutrition in Brazil during 2000−2015: A nationwide case-crossover study
Source: PLoS Med. 2019 Oct 29;16(10):e1002950. doi: 10.1371/journal.pmed.1002950 (PMC6818759; doi:10.1371/journal.pmed.1002950)
Supplement: S1 Text — (DOCX) [file pmed.1002950.s009.docx]

**S1 Text. Prospective analysis plan and modifications following comments from editors and reviewers**

**Date of commencing this research:** Feburary 5th, 2019.

**Research type:** A time-stratifed case-crossover study

**Research objectives**

1. To quantify the association between high ambient temperture (heat exposure) and hospitalizations due to undernutrition.

2. To explore whether the association between heat exposure and hospitalization for urdernnutrition varied across Brazilian five regions, population subgroups (i.e. sex and age), and different types of undernutrition.

3. To evaluate the hospitalization buden of undernutrition that are attributable to heat exposure during the study period.

4. To exploare whether there is a secular trend in the attributable burden from 2000 to 2015, given the global warming trend.

**Subjects**

Daily hospitalization cases recorded by Brazil’s National Unified Health System has been extracted for 1,814 cities in the hot season (city-specific four adjacent hottest months) during 2000−2015, as detailed by our previous study [1]. In this study ,we will focus on hospitalizations with ICD-10 codes (E40-E46) for undernutrition (<https://icd.who.int/browse10/2016/en>) as the primary dignosis.

**Exposure measurements**

We will obtain the daily minimum and maximum temperatures from a national meteorological dataset (0.25° × 0.25° resolution) developed by Xavier [2]. We will use the weather of city centre to represent each city as we did in previous study [1]. In this study, daily mean temperature (the average of daily minimum and maximum temperatures) during hot season will be used to represent heat exposure [1]. Hot season will be defined as the city-specific four adjacent hottest months during 2000-2015.

**Study design**

We will use a time-stratified case-crossover design. Briefly, for each hospital admission, the daily mean temperatures during the risk period (the admission date and several days before the admission) will be compared with those during the control periods in the same city. For each undernutrition case’s risk periods, control periods are the days at the same day of the week in the same calendar month. This design could adjust for time-constant confounders (e.g., age, sex, income, lifestyles) and time-dependent confounders (e.g., temporal trend and day of the week) [3, 4].

**Statistical Analyses**

**1. Descriptive analyses**

Basic characteristics of hospitalizations and tempertures in Brazil during the study period will be summarized, such as the number of undernutrition hospitalizations, the sex and age distributions, and the mean/median tempertures of selected cities during the study period.

**2. Heat-hospitalization associations**

2.1 Main model

We will use a conditional logistic regression to fit the relationship between ambient temperature and risk of undernutrition hospitalization, with equation as follows [5]:

$logit \left( P\left( case=1 in stratum i | Temp, Holiday \right) \right)=a_{stratum\left( i \right)}+cb\left( Temp \right)+dHoliday$

In this equation, a stratum is consisted of one case (case=1) and its three or four controls(case=0), the total number of stratums is equal to the number of hospitalization for undernutrition; $P(case=1 in stratum i |Temp, Holiday)$ is the conditional probability of being a case in the i^th^ stratum given two independent variables; $a_{straum(i)}$ represent the constant or intercept of stratum i (each stratum has an intercept);$Holiday$ is a binary variable indicating whether the date was a public holiday, in order to account for any changes in hospital use during public holidays; $cb\left( Temp \right)$ is the cross-basis function for daily mean temperature modelled by Distributed Lag Linear and Non-Linear Model [6].

We will perform some preliminary analyses to determine the parameters of the cross-basis function, such as the choice of linear or non-linear model, the maximum days of lag, the degree of freedoms. Both previous knowledge and Bayesian information criterion (BIC) will be used to select the optimal models.

2.2 Subgroup analyses

Stratified analyses will be performed by:

- sex (male and female)
- age groups (0−19, 20−39, 40−59, 60−79, and ≥80 years)
- five regions (North, Northeast, Central west, Southeast, and South)
- types of undernutrition [severe protein-energy malnutrition (PEM) (ICD-10 code E40-E43), moderate PEM (E44.0), mild PEM (E44.1), retarded development following PEM (E45), and unspecified PEM (E46)]

We will use random effect meta-regression to check the statistical differences between the subgroup effect estimates.

2.3 Sensitivity analyses

Sensitivity analyses will be performed to test the robustness of our findings. Potential sensitivity analyses includes:

- change the degrees of freedom in the $cb\left( Temp \right)$
- change the maximum lag days in the $cb\left( Temp \right)$
- adjust for relative humidity in the dataset of 193 cities with data on relative humidity

We will use fixed effect meta-regression to test whether the effect estimates from the main model is different from the effect estimates from alternative models.

**3. Estimate the attributable burden**

3.1 Estimate the total attributable burden

Due to limited city-specific sample size, we will use regional effect estimates along with city-specific tempertures to evaluat the attributable fraction and cases of undernutrition hospitaliation due to heat exposure. The deitailed procedure depends on the linearity of the relationship and choice of maximun lag days [7, 8].

We will estimate the attributable cases and attributable fractions (AFs) with 95% confidence interval for all 1,814 cities. We will report the agregated estimates for the whole nation, five regions, and different subgroups.

3.2 Evaluate the long-term trend of the AFs from 2000-2015

After getting the region-year-specific AFs, we will evaluate the long-term trend of the AFs. The trend will be evaluated by fitting the relationship between region-year-specific AFs and year using linear mixed effect model, only adjusted for the random effects of region. Similar method will also be used to evaluted the long-term trend of region-year-specific temperature.

Finally, we will fitted the relationship between region-year-specific AFs and mean temperatures using a linear mixed effect model, only adjusting for the random effect of region. This will tell us whether the trend of AF is related to the trend of temperature.

**4. Statistical software**

All analyses will be performed in R software (version 3.3.2). The R packages that will be used include “dlnm”, “survival”, “mvmeta”, “nlme”, “ggplot2” et al.

**Modification based on the comments from editors and reviewers:**

According to the suggestion of reviewers, we made three revisions:

1. We further stratified the age group of 0-19 years into two smaller groups: 0-4 years and 5-19 years.
2. We added a sensitivity analysis by including 5 or 6 city-specific hottest months as the hot season.
3. We repeated our main model analyses in cold season (city-specific 4 coldest months) and moderate season (city-specific months other than cold and hot season), in order to examine whether the effects of temperture rise on hospitalization for undernutrition will attenuate in cooler months.

**References**

1. Zhao Q, Li S, Coelho M, Saldiva PHN, Hu K, Arblaster JM, et al. Geographic, Demographic, and Temporal Variations in the Association between Heat Exposure and Hospitalization in Brazil: A Nationwide Study between 2000 and 2015. Environmental health perspectives. 2019;127(1):17001. Epub 2019/01/09.

2. Xavier AC, King CW, Scanlon BR. Daily gridded meteorological variables in Brazil (1980-2013). Int J Climatol. 2016;36(6):2644-59.

3. Janes H, Sheppard L, Lumley T. Case–crossover analyses of air pollution exposure data: referent selection strategies and their implications for bias. Epidemiology. 2005;16(6):717-26.

4. Janes H, Sheppard L, Lumley T. Overlap bias in the case‐crossover design, with application to air pollution exposures. Stat Med. 2005;24(2):285-300.

5. Zhao Q, Li S, Coelho MSZS, Saldiva PHN, Hu K, Huxley RR, et al. Temperature variability and hospitalization for ischaemic heart disease in Brazil: A nationwide case-crossover study during 2000–2015. Science of The Total Environment. 2019;664:707-12.

6. Guo Y. Hourly associations between heat and ambulance calls. Environ Pollut. 2017;220:1424-8.

7. Gasparrini A, Leone M. Attributable risk from distributed lag models. Bmc Med Res Methodol. 2014;14.

8. Hu K, Guo Y, Hu D, Du R, Yang X, Zhong J, et al. Mortality burden attributable to PM1 in Zhejiang province, China. Environ Int. 2018;121(Pt 1):515-22. Epub 2018/10/07.
